# Cost-Effectiveness of Difelikefalin for the Treatment of Moderate-to-Severe Chronic Kidney Disease-Associated Pruritus (CKD-aP) in UK Adult Patients Receiving In-Centre Haemodialysis

**DOI:** 10.3390/jcm14124361

**Published:** 2025-06-19

**Authors:** Kieran McCafferty, Cameron Collins, Imogen Taylor, Thilo Schaufler, Garth Baxter

**Affiliations:** 1 Barts Health NHS Trust, London E1 1BB, UK; 2 Initiate Consultancy Ltd., Alderton NN12 7LS, UK; 3 CSL Vifor, 9014 St. Gallen, Switzerlandgarth.baxter@viforpharma.com (G.B.)

**Keywords:** difelikefalin, chronic kidney disease, pruritus, dialysis, cost-effectiveness

## Abstract

**Background/Objectives**: CKD-associated pruritus (CKD-aP) is a serious systemic comorbidity occurring in patients with CKD. Despite the burden of CKD-aP, there are limited efficacious treatments available for its management; difelikefalin is the only approved treatment based on its efficacy and safety demonstrated in two clinical studies, namely KALM-1 and KALM-2. This study aimed to evaluate the cost-effectiveness of difelikefalin plus best supportive care (BSC) versus BSC alone when treating moderate-to-severe CKD-aP in patients receiving in-centre haemodialysis, from the perspective of the UK healthcare system. **Methods**: A de novo lifetime Markov health economic model was built to assess the cost-effectiveness of difelikefalin. The modelled efficacy of difelikefalin was based on data from KALM-1 and KALM-2 pooled at the patient level. The main efficacy driver was the total 5-D Itch scale score. Per-cycle probabilities of changing health states defined by CKD-aP severity were used to derive transition matrices; the model also estimated time-dependent annual probabilities of death and transplant for people on haemodialysis. An increased risk of mortality for modelled patients with very severe, severe, or moderate CKD-aP was applied. Health state utilities and management costs were based on published evidence. **Results**: Modelled patients treated with difelikefalin were estimated to have a reduced severity of CKD-aP. Consequently, difelikefalin plus BSC was associated with an increased life expectancy of 0.11 years per person and improved HRQoL compared with BSC alone. This translated to higher quality-adjusted life years, at 0.26 per person gained compared to BSC alone. Improved patient outcomes were achieved at an incremental cost of £7814 per person. **Conclusions**: Overall, at a price of £31.90/vial, difelikefalin was estimated to be a cost-effective treatment for moderate-to-severe CKD-aP at a willingness-to-pay threshold of £30,000/QALY, with conclusions robust to sensitivity analysis.

## 1. Introduction

### 1.1. Disease Background: Chronic Kidney Disease-Associated Pruritus

CKD-associated pruritus (CKD-aP) is a significant comorbidity that occurs commonly in CKD patients [1]. Compared to CKD patients without pruritus, CKD-aP patients have increased rates of diabetes, sleep disturbance, and depression; they are more likely to be hospitalised for infections; and they have an increased risk of mortality [1,2,3,4,5]. The severity of pruritus, as well as its location on the body, can vary over time in individual patients. In its most severe forms, CKD-aP can lead to a patient being completely restless during both the day and night, significantly reducing their health-related quality of life (HRQoL); this is particularly true when it appears as part of a symptom cluster with other CKD comorbidities [4,6]. It is thought that by treating one symptom, other symptoms experienced by people with CKD-aP, such as emotional and psychological distress, may also be alleviated [4].

An estimated 47% of CKD patients on haemodialysis (HD) in the UK experience moderate-to-severe CKD-aP [5,7]. Treating clinicians often underestimate the prevalence of pruritus in their patients. One recent study of CKD-aP patients in 21 countries found that 17% of those who were ‘always’ or ‘nearly always’ bothered by pruritus had not reported their symptoms to a healthcare professional [1].

CKD-aP is clearly burdensome, but the disease has few evidence-based treatments. Difelikefalin is the first and only approved drug for this condition currently. Best supportive care (BSC) may include creams and emollients, as well as off-label use of antihistamines, gabapentin, and pregabalin. However, these therapies are supported by limited and low-grade clinical evidence and are prescribed heterogeneously.

### 1.2. Difelikefalin and KALM Trials

Difelikefalin (Kapruvia^®^) is an agonist of kappa-opioid receptors (KORs). Opioid receptors play a role in regulating itch signals; KORs specifically are known to reduce itch. Difelikefalin activates KORs found on peripheral sensory neurons and immune cells and as such can help to reduce pruritus and the associated inflammation. Difelikefalin is peripherally restricted, meaning its effect is limited to KORs outside the brain or spinal cord [8,9,10].

The pivotal multicentre, randomised, placebo-controlled studies KALM-1 and KALM-2 (NCT03422653, NCT03636269) represent the largest clinical development programme assessing a treatment for CKD-aP worldwide [11,12]. These trials evaluated the efficacy and safety of 0.5 mcg/kg difelikefalin treatment for reducing itch intensity in patients with moderate-to-severe CKD-aP. In the studies, difelikefalin was administered after each HD session (three times a week). Each trial had a double-blind phase and an open-label extension (OLE) phase.

Following assessments and the publication of guidance from the National Institute of Health and Care Excellence (NICE) on 17 May 2023 and the Scottish Medicines Consortium on 12 February 2024, difelikefalin is now recommended as a treatment option for moderate-to-severe CKD-aP throughout the UK [13,14].

### 1.3. Aims

Cost-effectiveness analysis is a key component in the effective and equitable allocation of healthcare resources. This study aimed to describe the conceptualisation and parameterisation of a de novo cost–utility model investigating the cost-effectiveness of difelikefalin plus BSC versus BSC alone when treating moderate-to-severe CKD-aP in patients receiving in-centre haemodialysis (ICHD) from the perspective of the UK NHS.

## 2. Materials and Methods

### 2.1. Patient Population

The target patient population for cost-effectiveness analysis was adult patients receiving chronic ICHD in the UK NHS and experiencing moderate, severe, or very severe CKD-aP based on the definitions reported by Lai et al., 2017 [15]. This population is aligned with the licenced indication for difelikefalin and is henceforth referred to as ‘moderate-to-severe’.

### 2.2. Model Structure

A Markov model was developed including a set of disease states to represent the population of interest over time, with patients allowed to reside in only one of these disease states at any point. The model also defines the ways in which patients transition between the disease states and the probabilities of these transitions occurring during each time period.

**Figure 1:**
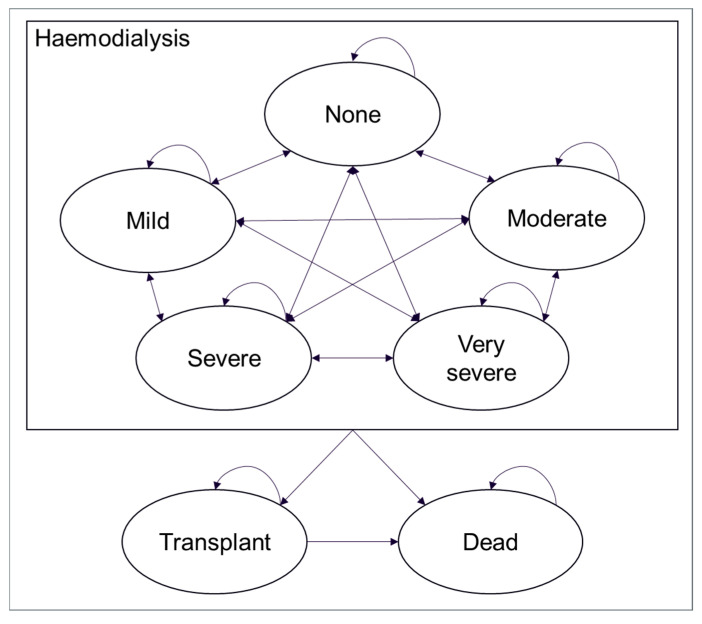
Model schematic.

The model incorporated five main health states, each defined by the level of pruritus severity, with none, mild, moderate, severe, and very severe, as defined by Lai et al., 2017 [15], based on scores from the 5-D Itch scale (categorised as follows: ≤8, none; 9–11, mild; 12–17, moderate; 18–21, severe; ≥22, very severe). Patients could only enter the model in the moderate, severe, or very severe health states; they could subsequently transition to ‘mild’ or ‘none’ as a result of treatment. From each state, they could also transition to transplant or death. Because renal failure is associated with CKD-aP, renal transplants were considered a definitive treatment for CKD-aP [16]. Figure 1 shows the structure of the model and how patients could transition between health states during each model cycle.

The model reflected UK clinical practice for CKD-aP at the time it was developed. It included firstly a ‘run-in’ period which incorporated initial treatment decisions and patients’ immediate response to this treatment based on the results of KALM-1 and KALM-2, followed by long-term extrapolation based on the OLE phases of the trials. Patients who did not achieve a clinically meaningful response during the run-in period and thus discontinued difelikefalin treatment progressed through the model at the same rate as the BSC arm. In the KALM trials, patients were not removed from treatment in this way, meaning it was not possible to specifically model outcomes for this patient group.

Patients responding to treatment with difelikefalin in the first 12 weeks could remain on treatment until transplant or death. Non-responders were assumed to discontinue difelikefalin and remain on BSC alone. Following the initial run-in period of three 4-week cycles, the model used 52-week cycles over a lifetime time horizon. Costs and benefits were both discounted at an annual rate of 3.5%, in line with the NICE methods guide [17].

### 2.3. Efficacy

The model estimated treatment efficacy using anonymised and pooled patient-level data from the KALM trials, with two additional Phase 3 supportive studies used to help define the characteristics of the model population at baseline. The specific population used to define baseline characteristics was the ‘all-difelikefalin-exposure’ cohort specified by Fishbane et al. in 2022 [12]. This comprised all participants who received at least one dose of difelikefalin for up to 64 weeks, from the placebo-controlled periods of KALM-1 and KALM-2 (if randomised to difelikefalin), the open-label extension period of these studies, or from 2 additional Phase 3 supportive studies (CLIN3101 for up to 52 weeks and CLIN3105 for up to 12 weeks) [12]. An overview of the baseline characteristics of this population is provided in Appendix A. CLIN3101 and CLIN3105 were open-label studies which evaluated the safety and tolerability of difelikefalin, and they were included to increase the sample size used to define characteristics at baseline; KALM-1 and KALM-2, on the other hand, also evaluated the efficacy of difelikefalin in the context of a randomised controlled trial. As such, transition matrices were derived using KALM-1 and KALM-2 only, as detailed below.

The main efficacy driver in the model was the total 5-D Itch scale score, a questionnaire assessing five dimensions of itch which is validated for use with patients with chronic pruritus, including those on HD, and is sensitive to changes in pruritus over time. This, alongside WI-NRS and Skindex-10, was one of three measures used to assess itch severity and itch-related HRQoL in KALM-1 and KALM-2. It was selected for use based on the results of a separate primary data collection study [18] which developed an algorithm mapping WI-NRS and 5-D Itch to EQ-5D-3L. In this analysis, mapping models including 5-D Itch performed better in terms of model fit and predictions than models including only a WI-NRS measure.

In the base case, the probability of losing or gaining health states in each cycle, estimated using the mean change in the total 5-D Itch scale scores from the baseline and stratified by the baseline severity of CKD-aP, was used to derive transition matrices. This approach aligns with the endpoints of KALM-1 and KALM-2, in which changes in itch were measured from baseline to the end of Week 12.

Clinical opinion was sought regarding the expected mean change in itch scores during the extrapolation period of the model for patients receiving placebo and difelikefalin. This was in the form of a modified Delphi panel with eight advisors from England and later on in an advisory board with four advisors from Scotland [19]. The advisors expected that the placebo effect would wane over time, in line with the natural progression of CKD-aP, and that difelikefalin would show no treatment waning effect. Additionally, a review of atopic dermatitis NICE technology appraisals was conducted to provide estimates on the long-term benefit of BSC treatments for pruritus; all three appraisals identified had used the same scenario, in which all clinical benefit for patients receiving BSC was lost by the end of Year 5.

As such, a waning effect was applied independently to each of the five core health states for the BSC treatment arm, which more conservatively assumed that patients returned to their baseline health state distribution by Year 10.

### 2.4. Mortality and Transplant Rates

The NICE guideline for renal replacement therapy (RRT) and conservative management, NG107, presents estimates for the probability of death and transplant for people on HD each year for up to 10 years after their first dialysis session; all subsequent years are assumed to have identical probabilities of death as Year 10. This is performed as part of a cost-effectiveness analysis comparing hemodiafiltration (HDF) to high flux HD [20]. The probabilities are based on an analysis of data from the UK Renal Registry (UKRR) from a cohort of UK adults who started RRT through HD between 2005 and 2014. Annual mortality rates of patients on HD from 2015 and 2019 were similar to those presented by the UKRR, indicating their analysis is still generalisable to present-day HD populations. The base case of this model therefore used the methods presented in NG107 to derive mortality and transplant rates. Please see Table A3 in Appendix A for further information.

Patients who received a transplant were assumed to follow rates of age-adjusted all-cause mortality found in UK life tables. The probability of age-dependent mortality was estimated based on the distribution of males and females in the KALM clinical trials.

Sukul et al., 2021 [5], used a Cox regression to compare all-cause mortality rates for CKD-aP patients with pruritus to patients who did not report pruritus. Their results showed CKD-aP to be an independent predictor of patient mortality. The hazard ratios found in their analysis—1.24 for patients extremely bothered by pruritus, 1.02 for patients very much bothered, and 1.11 for patients moderately bothered—are applied in the base case analysis of this model to implement an increased mortality rate in patients with very severe, severe, and moderate CKD-aP [5].

### 2.5. Health-Related Quality of Life

Health state utilities for the five core health states were informed by the SHAREHD trial, a UK randomised study in patients receiving ICHD [21]. Lee et al., 2005, studied HRQoL in patients with kidney failure with transplants compared to those without transplants, and the study was used to inform utility values for kidney transplant [22]. The scores derived for each health state are displayed in Table 1.

### 2.6. Healthcare Costs and Health-Related Quality of Life

Difelikefalin is administered following HD treatment using an intravenous bolus injection at a recommended dose of 0.5 micrograms/kg dry body weight [10]. Vials are used only once; as such, the number of vials required in the model was rounded to ensure unused fill volume was accounted for.

The model used UKKR data on the number of ICHD sessions undertaken by CKD-aP patients to derive an estimate of the number of dialysis sessions per patient per week. Because the UKKR estimates only showed the number of patients undergoing ICHD less than three times weekly, exactly three times weekly, and more than three times weekly, it was assumed that those who had less than three sessions per week had two and those who had more than three sessions per week of four. This gave a weighted frequency of 2.96 dialysis sessions per patient per week. The yearly cost of difelikefalin was therefore estimated at £4915.02 (at £31.90/vial).

Data from the previously mentioned utility mapping study on background CKD-aP treatments was used to estimate the average annual cost of BSC, with data from the BNF used for unit costs, dose, and pack size [18,23]. The mapping study did not separately consider patients with severe and very severe CKD-aP, as both populations were very small. As such, in the base case analysis, the resource use for these two populations was to be equal.

The weighted treatment cost for BSC was included in total treatment costs in the difelikefalin arm. The total weighted treatment cost of BSC was found to be lower than in the ‘moderate’ CKD-aP population than in the ‘mild’ and ‘none’ populations, something which was attributed to lower use of antidepressants in the moderate CKD-aP population. However, because difelikefalin is added to BSC, it was assumed that total treatment costs were equal for the moderate and mild CKD-aP populations.

Table 2 shows the total weighted treatment costs for each health state.

Annual health state costs comprised visits in primary care, hospitalisations, tri-monthly in-person patient reviews with a consultant nephrologist, and transplant and post-transplant costs. The base case did not include the cost of dialysis, as the corresponding adjustment in the risk of mortality led to an indirect increase in survival for the difelikefalin treatment arm. Appropriate unit costs were sourced from the National Cost Collection and Personal Social Services Research Unit [24,25].

In the KALM studies, ≥65% of adverse events (AEs) were mild or moderate in severity [12]. Because a systematic literature review failed to identify relevant or appropriate costs for these AEs, the base case analysis assumed all AEs to be costed as a single GP appointment (£33.19) [25]. In addition, given the small incremental incidence of AEs in patients treated with difelikefalin, which suggested that observed AEs were likely to be a feature of underlying disease, adverse event QALY loss was set to 0 for all AEs. Treatment discontinuations stemming from AEs were not modelled.

## 3. Results

### Base Case Analysis

Table 3 summarises the cost-effectiveness results of difelikefalin plus BSC versus BSC alone for the treatment of adults with moderate-to-severe CKD-aP over a lifetime model horizon (42 years). At a willingness-to-pay (WTP) threshold of £30,000/QALY and a cost of £31.90/vial, difelikefalin was estimated to be cost-effective.

Modelled difelikefalin-treated patients were estimated to have a reduced severity of CKD-aP. Consequently, the difelikefalin and BSC group had a higher life expectancy (0.11 years per person) and improved HRQoL compared with BSC alone, which translated to more quality-adjusted life years (QALYs, 0.26 per person) gained compared with BSC alone. Improved patient outcomes were achieved at an incremental cost of £7814 per person.

**Figure 2:**
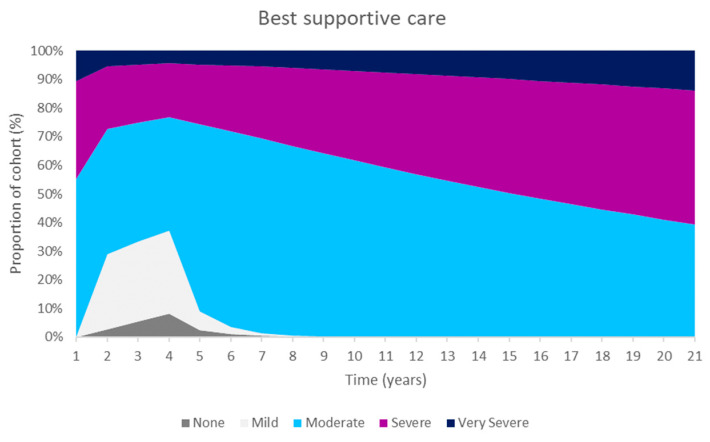

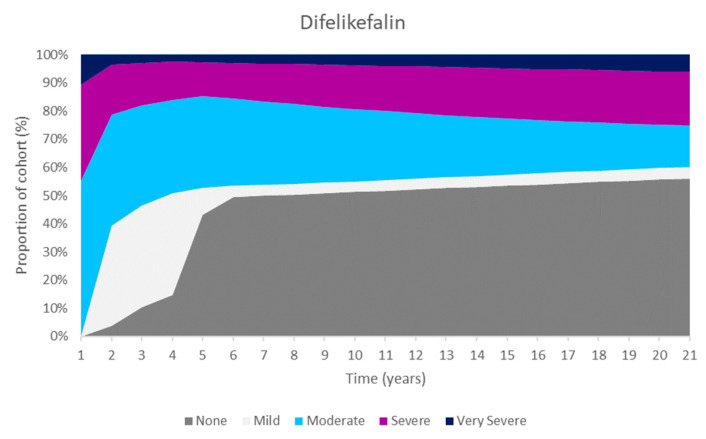
Markov traces.

These improvements were primarily as a result of an increase in the number of people in the ‘none’ CKD-aP health state. The Markov traces in Figure 2 display the distribution of patients across health states for difelikefalin and BSC. Difelikefalin was associated with reduced pruritus severity, which subsequently improved HRQoL and reduced the risk of mortality; this led to reductions in healthcare resource use and the use of concomitant medications.

**Figure 3:**
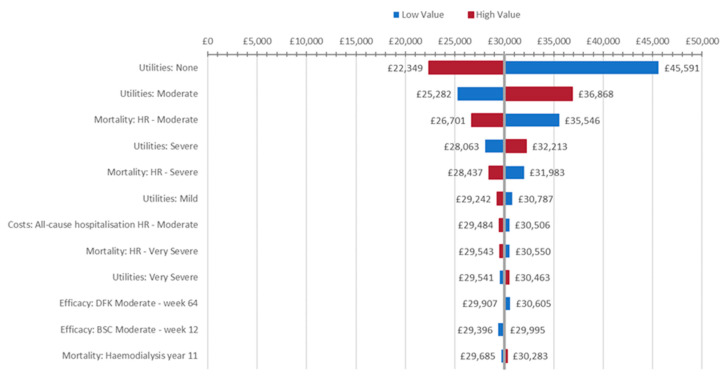
Tornado plot of DSA (impact ≥ £1000/QALY). Abbreviations: BSC, best supportive care; DFK, difelikefalin; DSA, deterministic sensitivity analysis; HR, hazard ratio.

The effect of varying individual model inputs on the uncertainty of the model was explored in two sets of analyses, beginning with deterministic sensitivity analyses (DSAs). Figure 3 presents the most influential model parameters according to these DSAs.

Changes in health state utility values, particularly that of the ‘none’ CKD-aP severity health state, were found to be the factor to which the cost-effectiveness of difelikefalin plus BSC was most sensitive.

**Figure 4:**
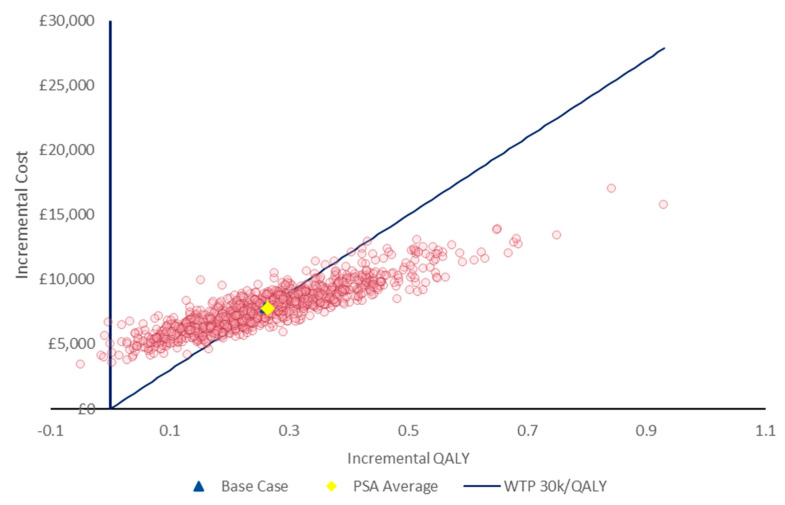
Cost-effectiveness plane from PSA (1000 simulations).

Probabilistic sensitivity analysis (PSA) was also performed; results for 1000 iterations are presented in Figure 4. The results of the PSA were consistent with the deterministic results, with incremental per-patient QALYs and costs being 0.26 and £7805, respectively. Difelikefalin was found to have a 48% probability of being cost-effective at a WTP threshold of £30,000/QALY, the upper end of NICE’s ‘standard’ threshold [26]. This probability increased to 79% and 90% at WTP thresholds of £40,000 and £50,000/QALY, respectively.

## 4. Discussion

The aim of this publication was to assess the cost-effectiveness of difelikefalin in addition to BSC versus BSC alone for adults with moderate-to-severe CKD-aP receiving ICHD from a UK payer perspective.

CKD-aP imposes a significant burden on patients’ HRQoL and to healthcare systems. CKD-aP, characterised by systemic pruritus, contributes to a myriad of complications, including poor sleep quality, depression, and increased mortality rates. Despite efforts to understand its pathophysiology and develop effective treatments, CKD-aP remains largely underdiagnosed and undertreated. With a lack of robust treatment recommendations and limited approved drugs, patients often rely on off-label interventions and BSC, leading to a high unmet need in this population. Addressing this issue requires greater awareness, research, and access to innovative therapies to alleviate the burden of CKD-aP on patients undergoing haemodialysis.

Moderate-to-severe CKD-aP is associated with a high unmet need, which treatment with difeliekfalin is likely to address. In KALM-1 and KALM-2, difelikefalin was shown to result in statistically significant and clinically meaningful improvements in both itch severity and itch-related HRQoL compared to a placebo over 12 weeks of treatment (5-D Itch scale: 52.1% vs. 42.3%; *p* = 0.01). This improvement was maintained in patients who continued difelikefalin treatment during the OLE period and emerged in patients who switched to difelikefalin from the placebo. By demonstrating its cost-effectiveness at a WTP threshold of £30,000/QALY, this model shows that difelikefalin can provide these benefits to a patient population with a high unmet need at a cost which does not unduly burden national healthcare systems.

The model outlined above employs a variety of sources. Health state utility values were based on published estimates from the SHAREHD trial. Various UK sources were used to identify costs, with additional model inputs taken from published NICE guidance in CKD and atopic dermatitis. Although inputs specific to CKD-aP would be preferable, these were not available, and the use of sources from closely related conditions should therefore be regarded as the best feasible option.

DSA and PSA were used to test the effect of varying model inputs and assumptions on the uncertainty of the model, with both finding the base case cost-effectiveness results to be robust. The use of other utility scores derived from an analysis of SHAREHD improved the cost-effectiveness of difelikefalin; difelikefalin was also more cost-effective in patients who had severe or very severe itch at baseline, demonstrating its ability to provide relief from disease symptoms from those patients in most need.

Markov models such as this one make an assumption that all patients in a disease health state are homogeneous, and as such, disease and HRQoL outcomes are determined only by the factors that define that health state (in this model, pruritus severity and the modality of renal replacement therapy). In reality, the causes of these outcomes are multi-faceted, and patients in each health state will be heterogeneous in many respects. Although this is a necessary simplification to facilitate economic analysis, this is nevertheless a strong analytical assumption and limitation. Furthermore, the extrapolation of outcomes far beyond the trial period of KALM-1 and KALM-2 to assess the cost-effectiveness over a lifetime horizon introduces significant uncertainty to the analysis. It should also be considered that there is no objective way to measure the intensity of itch, although the 5-D Itch scale has been validated for use in patients on HD with pruritus. Patient perception and self-reporting of itch may confound the estimated cost-effectiveness of difelikefalin, even though any confounding is likely to be present in both arms of the analysis.

## 5. Conclusions

In this analysis, difelikefalin was estimated to be a cost-effective treatment for moderate-to-severe CKD-aP at a WTP threshold of £30,000/QALY for costs up to £31.90 per vial. Difelikefalin has the potential to ameliorate the significant burden CKD-aP imposes on both patients and the healthcare system in the UK.

## Figures and Tables

**Table 1:** Health state utility scores.

Health State	Utility Score
None	0.744
Mild	0.726
Moderate	0.589
Severe	0.595
Very severe	0.595
Transplant	0.712

**Table 2:** Total weighted treatment costs by health state.

Health State	Best Supportive Care	Health State Weighted Cost (Annual) ^1^
None	£31.98	£3726
Mild	£42.48	£3753
Moderate	£42.48 *	£3891
Severe	£75.65	£4084
Very severe	£75.65	£4304

Notes: * BSC costs for moderate CKD-aP are adjusted to equal costs for mild CKD-aP; the very severe health state is assumed equivalent to the severe health state. ^1^ Health state costs include GP visits, hospitalisation, specialist visits (nephrologist), and haemodialysis. Abbreviations: BSC, best supportive care; CKD-aP, chronic kidney disease-associated pruritus.

**Table 3:** Cost-effectiveness results of difelikefalin versus BSC over 42 years.

	Difelikefalin + BSC	BSC	Incremental	ICER (£/QALY)
Total costs (GBP)	£31,516	£23,702	£7814	£29,995
Total life years (LY)	4.64	4.53	0.11	
Total QALYs	3.20	2.93	0.26	

Abbreviations: BSC, best supportive care; ICER, incremental cost-effectiveness ratio; LY, life year; QALY, quality-adjusted life year.

## Data Availability

No new data were generated or analysed in support of this research.

## Appendix A

Table A1 provides a summary of the baseline characteristics of the patients in the pooled all-difelikefalin-exposure cohort, as taken from Fishbane et al. 2022 [12], and used to define the characteristics of the model population at baseline [12].

**Table A1:** Baseline demographics and disease characteristics of pooled KALM-1, KALM-2, CLIN3101, and CLIN3105 population.

Characteristic	All-Difelikefalin-Exposure Cohort (N = 1306)
Age, mean (SD), years	58.3 (12.8)
Age ≥ 65 years, n (%)	425 (32.5)
Male, n (%)	767 (58.7)
Race, n (%)	
White	692 (53.0)
Black or African American	494 (37.8)
Asian	57 (4.4)
Other	58 (4.4)
Unknown or not reported	5 (0.4)
Dry body weight, mean (SD), kg	84.4 (21.5)
Time since diagnosis of ESRD, median (IQR), years	4.3 (5.4)
Duration of pruritus, median (IQR), years	2.6 (3.5)
Years on chronic HD, median (IQR), years	4.0 (5.2)
Prior anti-itch medications, n (%)	517 (39.6)
Most commonly used anti-itch medications, n (%)	
Diphenhydramine	321 (24.6)
Hydroxyzine	131 (10.0)
Hydrocortisone	30 (2.3)
Cetirizine	20 (1.5)
Clemastine	18 (1.4)

Abbreviations: ESRD, end-stage renal disease; HD, haemodialysis; IQR, interquartile range; SD, standard deviation.

Table A2 provides a summary of total per-patient disaggregated costs in the base case. Costs were calculated by the cost per year in each health state multiplied by the time spent in that health state.

**Table A2:** Base case disaggregated costs.

	Difelikefalin + BSC	BSC	Incremental
**Treatment costs (£31.90)**	**£7818**	**£138**	**£7680**
**Adverse events**	**£17**	**£37**	**−£20**
**Management costs**	**£23,681**	**£23,528**	**£153**
None	£4585	£123	£4463
Mild	£788	£441	£347
Moderate	£3038	£6246	−£3209
Severe	£1552	£2783	−£1231
Very severe	£384	£674	−£290
Transplant	£13,334	£13,261	£73

Abbreviations: BSC, best supportive care.

Table A3 provides a summary of the QALYs and LYs associated with difelikefalin plus BSC and BSC alone in the base case.

**Table A3:** Base case disaggregated quality-adjusted life year and life years.

	Difelikefalin + BSC	BSC	Incremental
**Life years (total)**	**4.64**	**4.53**	**0.11**
None	1.23	0.03	1.20
Mild	0.21	0.12	0.09
Moderate	0.78	1.61	−0.82
Severe	0.38	0.68	−0.30
Very severe	0.09	0.16	−0.07
Transplant	1.95	1.94	0.01
**QALYs (total)**	**3.20**	**2.93**	**0.26**
None	0.92	0.02	0.89
Mild	0.15	0.09	0.07
Moderate	0.46	0.95	−0.49
Severe	0.23	0.41	−0.18
Very severe	0.05	0.09	−0.04
Transplant	1.39	1.38	0.01

Abbreviations: BSC, best supportive care; QALY, quality-adjusted life year.

Mortality and transplant rates in the base case were modelled using the methods presented in NG107 [20]. The resulting probabilities are summarised below in Table A4.

**Table A4:** Probability of death and transplant each year post-initiation of HD used in model.

Year	Probability of Death	Probability of Transplant
1	0.187	0.039
2	0.140	0.050
3	0.144	0.058
4	0.156	0.060
5	0.166	0.058
6	0.170	0.051
7	0.188	0.049
8	0.201	0.030
9	0.187	0.030
10	0.200	0.017
11+	0.200	0.000
